# Machine learning reveals X chromosome transcriptional signatures that classify systemic lupus erythematosus in females

**DOI:** 10.1186/s41927-026-00669-1

**Published:** 2026-06-30

**Authors:** Lachlan G. Gray, Aiden Telfser, Hannah Hu, Alisa Kane, Elissa K. Deenick, Tri Giang Phan, Sara Ballouz

**Affiliations:** 1 Precision Immunology Program, Garvan Institute of Medical Research, Sydney, NSW Australia; 2 St Vincent’s Healthcare Clinical Campus, School of Clinical Medicine, Faculty of Medicine and Health, UNSW Sydney, Sydney, NSW Australia; 3 Department of Immunology, Allergy and HIV, St Vincent’s Hospital, Sydney, NSW Australia; 4 Kirby Institute, Faculty of Medicine and Health, UNSW Sydney, Sydney, NSW Australia; 5 School of Computer Science and Engineering, Faculty of Engineering, UNSW Sydney, Sydney, NSW Australia

**Keywords:** Machine learning, Systemic lupus erythematosus, X chromosome inactivation, Single-cell transcriptomics

## Abstract

**Background:**

Systemic lupus erythematosus (SLE) has a significant female bias; however, it remains unclear whether X-chromosomal dysregulation increases the risk in females. Furthermore, while the role of X-linked genes in SLE pathogenesis is recognised, the analysis of genetic risks in SLE has largely focussed on autosomal genes.

**Methods:**

Here, we analysed a public single-cell RNA sequencing (scRNA-seq) dataset of peripheral blood mononuclear cells using machine learning to test whether X-linked gene expression classifies female SLE in a cell type-specific manner. Using pseudobulked expression profiles from 1.1 million cells across 228 female donors, we trained an ensemble of classifiers on all X-chromosomal genes or a literature-derived set of SLE-associated genes.

**Results:**

In CD4^+^ and CD8^+^ T cells, models based on X-chromosomal genes achieved classification performance comparable to SLE gene models and were significantly enriched for XCI escape genes, with IL2RG, CD40LG, and TKTL1 emerging as key features. When applied to an independent paediatric–adult SLE cohort, CD8^+^ T-cell models retained a consistent performance, indicating a stable and reproducible X-linked signature in this subset. Flow cytometry revealed a modest increase in IL2RG protein expression in SLE T cells, consistent with partial escape from XCI in these cells.

**Conclusions:**

These findings demonstrate that X-linked transcriptional programs, enriched for known XCI escape genes and concentrated in activated T cells, encode a reproducible transcriptional signal of female SLE and provide a framework for dissecting the contributions of sex chromosomes to autoimmunity.

**Supplementary Information:**

The online version contains supplementary material available at https://doi.org/10.1186/s41927-026-00669-1.

## Background

Systemic lupus erythematosus (SLE) is a prototypical autoimmune disease characterised by antinuclear antibodies (ANAs), increased type 1 interferon signalling (IFN), and disrupted clearance of apoptotic debris [1]. Early diagnosis is critical yet remains challenging because of the heterogeneous clinical presentation and symptoms that overlap with other inflammatory diseases. Notably, these complexities lead to an average diagnostic delay of 3 years [2].

SLE, like many autoimmune diseases, shows a marked female predominance (9:1), disproportionately affecting young women, in whom it is the leading cause of mortality [3, 4]. With the advent of single-cell RNA sequencing (scRNA-seq), studies of SLE have identified differences in immune cell composition, cell type specific patterns of gene expression, and highlighted novel pathogenic mechanisms, such as interferon-driven activation of B and T cells and the expansion of pathogenic myeloid subsets [5–8]. Despite these advances, few scRNA-seq studies have examined how sex differences, particularly the role of the X chromosome, shape immune dysregulation in patients with SLE.

In SLE, sex differences are evident in incidence and severity, with males displaying a lower incidence but more severe lupus nephritis [9]. Although the mechanisms underlying the female bias in SLE are incompletely understood, sex hormones and sex chromosomes are likely to play critical roles. For instance, sex hormones, including fluctuating oestrogen and progesterone levels, have been linked to increases in disease activity, known as flares [10]. Sex chromosomes have also been linked to increased risk, with X chromosome aneuploidies such as Klinefelter’s syndrome (XXY) and Trisomy X (XXX) conferring a 14-fold and 2.5–3.5-fold increased risk, respectively [11–13]. These studies suggest that X-linked dosage, particularly genes escaping X chromosome inactivation (XCI), contribute to SLE risk. One prominent example is toll-like receptor 7 (*TLR7*). TLR7 is biallelically expressed in naïve B cells, monocytes and plasmacytoid dendritic cells (pDC) from females and XXY males [14]. However, *TLR7* likely represents one of several X-linked immune genes whose dosage and regulation may contribute to sex-biased immune phenotypes in patients with SLE. A possible mechanism underlying XCI escape is disruption of the epigenetic maintenance of the inactive X chromosome, a process initiated and coordinated by the long non-coding RNA *XIST* [15]. In SLE, epigenetic maintenance of the inactive X chromosome appears dysregulated in lymphocytes, as B cells and T cells from patients exhibit incomplete XCI during differentiation from naïve to effector states [16–19]. Consistent with this lymphocyte specificity, computational analyses of single-cell RNA-seq and chromatin accessibility datasets identify stronger patterns of XCI escape in lymphocytes compared with myeloid cells [20]. Furthermore, recent work demonstrates that *XIST* functions both as a female-specific autoantigen and as a ligand for TLR7, thereby triggering interferon production in pDCs [21, 22].

While machine learning (ML) approaches have been used to classify SLE and identify key transcriptomic features, most have not considered sex-based differences, as male and female data are often combined [23–25]. Additionally, allele-specific expression, XCI, and X-linked escape were not considered because of technical difficulties in these analyses at single-cell resolution. In this study, we combined scRNA-seq with ML to test whether X-linked gene expression alone is sufficient to discriminate female patients with SLE in a cell type–specific manner. Using pseudobulked expression profiles, our models accurately classified SLE patients within CD4^+^ and CD8^+^ T cells and generalised to an independent paediatric–adult cohort. Notably, models trained on X chromosome genes performed comparably to literature-supported SLE gene sets and were significantly enriched for known XCI escape genes. Together, these results indicate that cell type–specific dysregulation of X-linked transcription encodes a stable discriminative signal for female SLE. Critically, our findings provide a conceptual advance over previous genome-wide studies by establishing that the X chromosome alone contains a concentration of signal that accurately classifies female SLE patients from controls. This study provides a framework for characterising the unique transcriptional landscape of the X chromosome that drives the female-specific SLE signature, moving beyond a descriptive analysis towards a classification model of X chromosome dysregulation.

## Methods

### Single cell RNA sequencing data collection

The scRNA-seq dataset generated by Perez et al. (2022) [8] was downloaded from the CellxGene database [26] as an ‘.h5ad’ file. The downloaded dataset included cells that passed quality control filters. Importantly, technical variation arising from batch effects were removed using COMBAT. Finally, cell types were annotated using canonical marker gene expression and confirmed by antibody staining.

### Data processing

The dataset was read into Python using the Scanpy package [27]. To focus this analysis on XCI in females, male samples were removed. To reduce flare-associated transcriptional noise, only samples indicated as controls or managed were included. Finally, only individuals with European or Asian ancestry were selected, as African American, Hispanic, and Latin American comprised too few individuals. The data was then read into R using the Seurat package for dimensionality reduction, clustering, and projection with Uniform Manifold Approximation and Projection (UMAP) [28, 29].

### Pseudobulking by cell type and gene set

Across cell types we aggregated batch corrected counts into a pseudobulked gene expression matrix. We used a pseudobulking approach to mitigate several inherent challenges of scRNA-seq data. Specifically, pseudobulking addresses the high level of sparsity and ‘dropout’ events characteristic of individual cell profiles, which can introduce noise into ML models. Furthermore, by aggregating cells within each biological replicate, we account for the hierarchical structure of the data, ensuring that the samples are independent, and the model learns features representative of patient-level variation rather than intra-sample cell-to-cell stochasticity.

We evaluate our ML models on two gene sets: all X chromosome genes (1,889 genes) and a positive control set of experimentally validated SLE genes (1,883 genes) obtained from the DisGeNet database [30].

### Training and testing data partitions

We constructed training and testing indexes using the Scikit-learn ‘train_test_split’ function with a training size of 80% and a test size of 20% [31]. The proportions of each class (control and disease) and ancestry (European and Asian) were maintained by incorporating each into the stratify parameter. This process was repeated ten times to account for the heterogeneity among patients with SLE. The training and testing gene expression values were z-score scaled prior to model training with the Scikit-learn ‘StandardScaler’. To prevent data leakage and artificial inflation of model performance, this was applied separately to each set.

### Boruta and ElasticNet feature selection

To reduce dimensionality and improve model performance, we applied the feature selection methods Boruta and ElasticNet [32, 33]. Boruta uses a random forest wrapper to retain features with consistently high importance, while ElasticNet applies regularised regression to remove redundant features and shrink the coefficients of correlated features. Feature selection was performed independently within each training partition. Features selected in the training set were then used to filter the corresponding testing set. This process was repeated for each of the ten data partitions to ensure that no information from the test set influenced feature selection. We compared features selected by each method individually, as well as their intersection and combination, and found that using both together improved model performance across cell types. To measure and assess potential correlated features and their impact, we calculated the pairwise Spearman correlation for the final selected features across all cell-type matrices and cross-validation splits.

### Training machine learning classifiers

To evaluate the performance of different machine learning classifiers, we tested five algorithms: logistic regression (logit), random forest (RF), support vector machine (SVM), gradient boosting machine (GBM), and multilayer perceptron (MLP). Each model was implemented using the scikit-learn Python library and optimised using the ‘GridSearchCV’ function with 10-fold cross-validation repeated three times [31]. When supported by the algorithm, models were trained with class_weight=“balanced” to account for class imbalance. For logit, models were trained using ‘LogisticRegression’ with the ‘saga’ solver and ‘elasticnet’ penalty. The regularisation strength (C) and L1 ratio (l1_ratio) were tuned across logarithmic scales (C = [0.01, 0.1, 1, 10], l1_ratio = [0, 0.5, 1]). RF classifiers were trained using RandomForestClassifier() and tuning n_estimators = [100, 200, 300], max_depth = [5, 10, 15], min_samples_split = [2, 5, 8], and max_features = [‘sqrt’, 0.3]. SVM was implemented with ‘SVC’, and the kernel choice and regularisation strength were tuned (kernel = [‘linear’, ‘rbf’, ‘poly’, ‘sigmoid’], C = [0.1, 1, 10, 100, 1000]). GBM used ‘GradientBoostingClassifier’ with tuning of learning_rate = [0.1, 0.05, 0.01], n_estimators = [100, 200, 300], max_features = [‘sqrt’, 0.3], and max_depth = [3, 5, 7]. MLP were implemented with ‘MLPClassifier’ and tuned for hidden_layer_sizes = [(50,), (100,), (50, 50), (100, 100)], solver = [‘sgd’, ‘adam’], alpha = [0.001, 0.01, 0.1], and learning_rate = [‘constant’, ‘invscaling’, ‘adaptive’].

### Soft voting ensemble classifier

To take advantage of the diverse strengths of each ML algorithm, we used an ensemble classification approach with the scikit-learn VotingClassifier() function. Rather than relying on a single algorithm, which exhibit specific biases, an ensemble combines the outputs of several models. We implemented soft voting to integrate these individual results. In soft voting, each model calculates the probability that a sample belongs to the SLE group. The ensemble then averages these probabilities to reach a final decision. This approach is analogous to a consensus of experts, reducing the risk of making an incorrect classification based on a single model.

### Evaluating model performance

Class labels were assigned to the testing data and classification probabilities calculated to evaluate model performance. Imbalanced datasets were accounted for by modifying assignment thresholds from the typical 0.5 with Youden’s J statistic, a metric that calculates thresholds minimising differences between the true positive rate (TPR) and false positive rate (FPR) from a receiver operating curve (ROC) [34]. Performance metrics are then calculated from the updated classification probabilities. The Matthews Correlation Coefficient (MCC) was selected as the primary performance metric because it provides a more balanced and reliable assessment of classification performance in imbalanced datasets than Accuracy or F1-score [35]. In our cohort, the uneven distribution of SLE cases versus controls means that high accuracy can be achieved by simply classifying the majority class. Unlike the F1-score, which ignores the True Negative rate, MCC considers all four quadrants of the confusion matrix (True Positives (TP), True Negatives (TN), False Positives (FP), and False Negatives (FN)). MCC ranges from −1 to + 1 and a high MCC score indicates that the model is performing well in both classes, ensuring that our X-linked signatures are truly discriminative of disease status rather than reflecting class imbalance [36].

The non-parametric Wilcoxon rank-sum test was employed to compare MCC across groups of interest [37]. When testing across cell types, *p* values were adjusted to false discovery rates (FDR) to account for multiple testing and reduce the FPR [38].

### Shapley additive explanations (SHAP)

The relative contribution of each feature to ensemble model classification was evaluated using Shapley additive explanations (SHAP) [39]. Within SHAP, an explainer object is created from KernelExplainer() by providing a function that calculates classification probabilities for a given model and training dataset. The explainer() function then calculates shapley values from the testing dataset.

### Additional independent SLE dataset

To evaluate model generalisability, we downloaded the Nehar-Belaid et al. 2020 [7] scRNA-seq dataset (GSE135779) from the National Center for Biotechnology Information (NCBI) Gene Expression Omnibus (GEO) [40]. This dataset includes 56 donors: 40 SLE patients (33 paediatric and 7 adult) and 16 healthy controls (11 paediatric and 5 adult) comprising 331,402 PBMCs. The data was read into R with Seurat and low-quality cells removed using the R package ddqcR [41]. Multiple cells encapsulated in the same droplet, known as doublets, were identified and removed using scDblFinder [42]. The data then underwent dimension reduction, clustering, and UMAP using Seurat [28]. Cell type annotation was performed using CellTypist, an automated model trained on scRNA-seq data from immune cells identified in 20 tissues across 19 studies. CellTypist was run with the ‘Immune_All_Low’ model and ‘majority voting’, where predictions were refined by choosing the dominant cell type in each cluster [43]. The annotated cell type names were modified to match those in the Perez et al., 2022 dataset [8]. As sex was missing from the metadata, sex was predicted using a hierarchical clustering approach. First, normalised counts for female specific *XIST* and male specific *RPS4Y1* were pseudobulked with the Seurat AggregateExpression() function to sum expression counts across individuals. Counts were then scaled, and Euclidean distances between genes calculated with the R dist() function and hierarchical clustering with the centroid method with hclust(). Clusters were obtained by cutting the dendrogram into k = 2 groups. Predicted females were then evaluated by confirming the higher expression of *XIST*. Males were then removed from the analyses. Within adult and paediatric samples, the data was pseudobulked as above, and SLE status was classified by the ensemble models for matching cell types.

### Flow cytometry validation of IL2RG expression

To validate IL2RG protein expression, peripheral blood mononuclear cells (PBMCs) were analysed by flow cytometry from an independent cohort of 10 female patients with SLE and 10 healthy female controls. This experiment was designed to validate the direction and cell-type specificity rather than to provide precise effect size estimates. Venous blood samples were collected at St Vincent’s and Liverpool Hospitals (Sydney, Australia), and PBMCs were isolated by Ficoll density-gradient centrifugation and cryopreserved until use.

Thawed PBMCs were stained with a panel of fluorochrome-conjugated antibodies including Brillant Violet 421 CD4 (clone OKT4), Brillant Violet 605 CD8 (clone SK1), and Phycoerythrin IL2RG/CD13 (clone TUGh4), together with Ghost Red 710 viability dye. Cells were acquired on a BD LSR Fortessa and analysed using FlowJo (v10.10). T cells were identified by standard forward and side scatter gating for lymphocytes, followed by exclusion of dead cells, and then gating on CD4^+^ and CD8^+^ populations. IL2RG expression was quantified as geometric mean fluorescence intensity (MFI) within each subset. Statistical analyses were performed in R. Differences in IL2RG expression between SLE and control cohorts were assessed using the Wilcoxon rank-sum test.

## Results

### Data curation

**Fig. 1:**
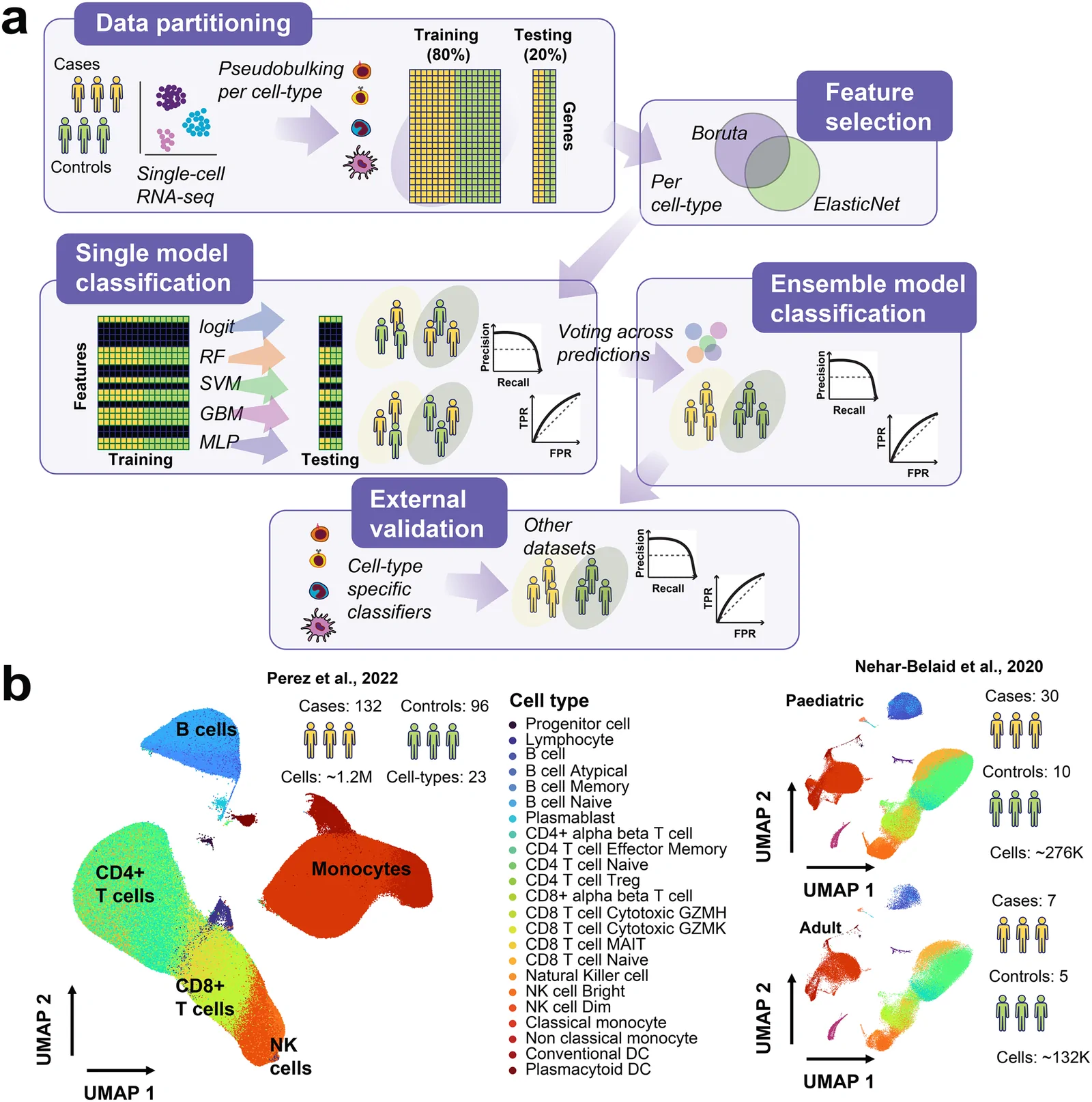
Overview of the study. (**a**) Schematic representation of the ML workflow used to classify SLE status from gene expression. Gene expression profiles were generated from pseudobulked scRNA-seq data, with 105 SLE cases and 79 controls included for model training (80%) and 27 cases with 20 controls reserved for model testing (20%) [8]. Feature selection was performed using Boruta and Elastic net to identify the most informative genes distinguishing cases from controls. Features selected by both methods were included in subsequent analysis. Multiple classifiers, including logistic regression (logit), random forest (RF), support vector machine (SVM), gradient boosting machine (GBM), and multilayer perceptron (MLP), were trained individually and their outputs combined into an ensemble classifier with soft voting. (**b**) UMAP projection of 1,116,410 PBMCs from 228 donors, clustered and annotated into 23 immune cell types, and the validation dataset of 52 cases and controls split into paediatric and adult cohorts

In this study, we assessed the classification performance of X chromosome genes using an ensemble ML framework (Fig. 1a). We began with a publicly available scRNA-seq dataset of PBMCs from 261 donors (162 SLE patients and 99 healthy controls) comprising 1,263,676 cells [8]. To focus our analysis on XCI, all males were excluded from the dataset. Additionally, flare-associated transcriptional noise was avoided by excluding patients experiencing disease flares. To reduce ancestry-related confounders, individuals with Hispanic, Latin American, or African American ancestry were removed as these ancestries comprised too few individuals. The remaining 1,116,410 cells from 96 controls (24 Asian and 72 European) and 132 SLE patients (70 Asian and 62 European) were included in downstream analysis. We used the 23 immune cell annotations from the original work, as these were determined by marker gene expression and confirmed by antibody labelling of cell surface proteins [8]. To account for within-individual dependence and reduce data sparsity, we pseudobulked batch-corrected gene expression by aggregating counts for each cell type per individual. Following pseudobulking, we trained five ML classifiers using cell type-specific gene expression. Feature selection was performed prior to training, and the classification models were evaluated on held-out test sets. Classification probabilities from each model were combined into an ensemble classifier, a strategy that improved robustness over individual models. Model generalisability was then evaluated by classifying an independent scRNA-seq dataset from Nehar-Belaid et al. [7]. This dataset includes 56 donors: 40 SLE patients (33 paediatric and 7 adult) and 16 healthy controls (11 paediatric and 5 adult) comprising 331,402 PBMCs. Sex was inferred from the expression of *XIST* (female) and *RPS4Y1* (male), and male samples were excluded. After quality control filtering, the validation set contained 52 female donors: 15 controls (10 paediatric and 5 adult) and 37 SLE cases (30 paediatric and 7 adult) comprising 310,310 cells (Fig. 1b). This framework allowed us to test whether X-linked features provide classifying power beyond established immune signatures.

### X chromosome models classify SLE in CD4^+^ and CD8^+^ T cells

**Fig. 2:**
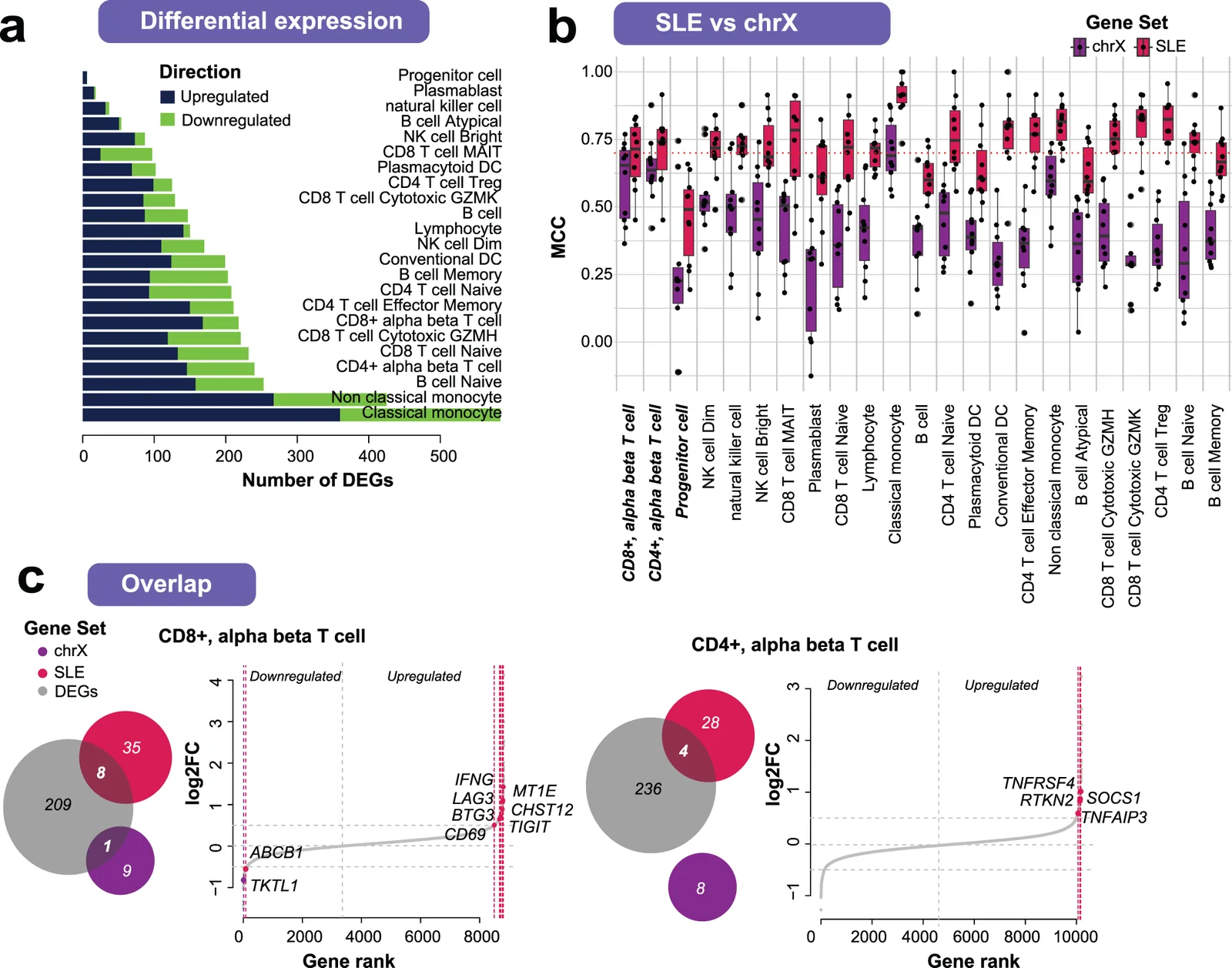
Performance of machine learning models. (**a**) Differential expression results across all cell-types ordered by number of genes. (**b**) Classification performance for SLE and X chromosome gene sets. Each boxplot shows Matthews correlation coefficient (MCC) values across cross-validation splits (Wilcoxon rank sum test with FDR correction, FDR > 0.05). Comparable classification performance to those trained on SLE-associated genes across ten immune cell types are italicised. (**c**) Overlap of features with DEGs from CD8^+^ T cells and CD4^+^ T cells. The gene ranks are the rank ordered importance scores assigned by the differential expression analysis using the log2FC and FDRs. Genes ranked near 1 are downregulated, and those near the end of the list are upregulated. The genes highlighted are those selected as features in the models and are DEG, indicating their relative rank in the DEG lists

Traditional studies perform differential expression analysis on gene expression data by comparing cases to controls. In this study we applied a ML framework to explore potentially more complex and non-linear interactions between genes that classify SLE cases versus controls. First, to benchmark the machine learning approach against standard methods, we performed differential expression analysis on pseudobulked data using the edgeR package [44]. After removing lowly expressed genes, we fitted a generalized negative binomial model with batch and age as covariates. After filtering for significant differentially expressed genes (DEGs) (FDR < 0.05; |logFC| >0.5), we observe that most DEGs were upregulated in SLE compared to controls (Fig. 2a).

We then applied our ML framework to each cell-type. Pseudobulked expression profiles were filtered for X chromosome genes (*N* = 1,889) or a set of literature supported SLE-associated genes from the DisGeNET [30] database (*N* = 1,883) (Additional file 1: Table S3). We reasoned that models trained on X chromosome genes (chrX) that displayed comparable performance to models trained on SLE-associated genes (SLE) would reveal whether X-linked expression is sufficient to classify SLE. To reduce the feature space and improve model interpretation, we performed feature selection with Elastic Net and Boruta and took the overlap of features selected by both methods [32, 33]. Across ten data partitions, the average number of features selected in chrX and SLE models was 4.43 ± 4.14 and 17.15 ± 18.25, respectively, reflecting substantial inter-partition heterogeneity consistent with the known molecular heterogeneity of SLE. After feature selection, we ran classifiers with increasing complexity: logistic regression (logit), random forest (RF), support vector machines (SVM), gradient boosting (GBM), and multilayer perceptron (MLP). Outputs from individual models were then combined into an ensemble classifier with soft voting to improve robustness. We trained each ML classifier using an 80:20 train-test split, stratified by disease status and ancestry, and repeated over ten data partitions. Performance was evaluated using Matthew’s Correlation Coefficient (MCC), a metric that ranges from −1 to + 1 and is robust in imbalanced datasets [35, 36]. Comprehensive performance metrics across all data splits, models, gene sets, and cell types including MCC, area under the curve (AUC), area under the precision recall curve (AUPRC), and weighted F1-score are reported in (Additional file 1: Table S1).

While standard differential expression analyses (Fig. 2a, Additional file 1: Table S4-5) identified a broad list of significantly dysregulated genes, our ensemble ML framework provides a distinct analytical advantage by identifying co-functional or co-regulated sets of features. Unlike differential expression, which compares the expression levels of genes in isolation, the combination of Boruta and ElasticNet feature selection methods identifies a minimal and non-redundant group of features that collectively function to classify SLE.

We then compared ensemble models trained on chrX and SLE genes to identify models with similar classification performance. In three cell types, chrX models achieved comparable classification to models trained on SLE-associated genes (Wilcoxon rank sum test with FDR correction, *p* > 0.05). These included CD4^+^ T cells, CD8^+^ T cells, and CD34^+^ Progenitor cells (Fig. 2b). However, Progenitor cells represented a very small fraction of the dataset, and their performance was unstable, with the 95% confidence interval for MCC ranging from 0.05 to 0.44, overlapping chance-level performance. Given the low abundance and lack of robust classification power, progenitor cells were excluded from further analysis. In contrast, CD4^+^ and CD8^+^ T cells showed consistently strong performance with 95% CI MCC values ranging from 0.55 - 0.73 and 0.49 - 0.70, respectively. We then asked whether X chromosome features selected across the ten partitions were enriched for known escape genes (Additional file 1: Table S2). Using all X-linked genes as the background, features selected in CD4^+^ T cells (*N* = 6) and CD8^+^ T cells (*N* = 9) were found to be significantly enriched for XCI escape genes (χ^2^ test, *p* < 0.01). Overlap with differential expression results showed that interleukin 2 receptor gamma (*IL2RG*) was significantly upregulated in CD4^+^ (logFC = 0.22; FDR < 0.001) and CD8^+^ T cells (logFC = 0.24; FDR < 0.01), whereas transketolase like 1 (*TKTL1*) was significantly downregulated in CD8^+^ T cells (logFC = −0.82; FDR < 0.01) (Fig. 2C). The absence of statistically significant changes for the remaining features highlights the utility of our ML approach, a framework that leverages subtle, multigene expression patterns to construct classification decision boundaries.

Given the lack of detailed medication metadata in the public cohort, we performed a genome-wide correlation analysis to ensure our top X-linked predictors were not capturing transcriptional signatures driven by standard SLE treatments, such as systemic corticosteroids. The selected features lacked significant correlation with canonical glucocorticoid-response genes. Rather, the Spearman correlation analysis revealed that selected X chromosome features were strongly correlated (|*r*| >0.7) with related inflammatory genes. For instance, in CD4+ T cells, the top X-linked predictor *CD40LG* was correlated with interleukin-7 receptor (*IL7R)*, an autosomal regulator of T-cell survival and memory. Similarly, in CD8+ T cells, *IL2RG* was correlated with a cytotoxic and activation signatures, including *CD2*, interleukin-32 (*IL32*), and granzyme M (*GZMM*). This association between X-linked features and canonical markers of T cell activation suggests that the models successfully captured networks reflecting the underlying disease dysregulation rather than confounders such as medications or demographic variables.

Overall, the strong performance of CD4^+^ T cells and CD8^+^ T cells is consistent with evidence implicating these cell types in SLE pathogenesis. CD4^+^ T cells contribute to the pathogenesis of SLE by providing help to autoreactive B cells and through the secretion of inflammatory cytokines. Furthermore, abnormalities in T cell receptor (TCR) signalling pathways lower the activation threshold in SLE to promote autoreactivity [45]. CD8^+^ T cells, in contrast, often display reduced cytotoxic capacity in SLE, a defect linked to increased susceptibility to infection, one of the leading causes of mortality in SLE [46]. Notably, memory and effector CD4^+^ and CD8^+^ T cells, but not naïve subsets, showed robust classification performance, consistent with reports that XCI escape signatures become more detectable following antigen-driven T-cell differentiation (Fig. 2b) [17, 19, 47]. This suggests that T-cell activation states may modulate X-linked transcription in ways that are informative for distinguishing SLE from controls.

### X chromosome models show strong performance across classical and non-classical monocyte lineage

While our primary analysis focused on cell types where chrX models showed comparable performance to SLE models, the monocyte lineage demonstrated strong MCC scores within the chrX models even though the SLE models outperformed them (Fig. 2a). In Classical monocytes, the SLE model significantly outperformed the chrX model (Mean MCC: 0.90 ± 0.10 vs. 0.60 ± 0.16), but the X-linked model alone retained robust classification value. Interestingly, these classification scores were partially retained as these cells matured into Non-classical monocytes (Mean MCC: 0.53 ± 0.11, compared to the SLE model at 0.83 ± 0.12). Classical monocytes (CD14^++^CD16^-^) comprise approximately 90% of circulating monocytes and display a highly inflammatory phenotype due to their role in migrating into inflamed tissues. In SLE, classical monocytes differentiate into mature non-classical monocytes (CD14^low^CD16^++)^, which phagocytose immune complexes and contribute to the initiation and progression of the disease [48, 49]. Inspection of the features selected in the monocyte chrX models revealed an overlap of known XCI escapees, including complement factor properdin (*CFP*), TIMP metallopeptidase inhibitor 1 (*TIMP1*), and iduronate 2-sulfatase (*IDS*). The immunological roles for these genes, particularly *CFP* and *TIMP1*, suggests that X-linked transcriptional dysregulation extends into the innate immune compartment and may contribute towards monocyte-driven pathogenesis in SLE.

### Shapley additive explanations reveal key features driving model outputs

**Fig. 3:**
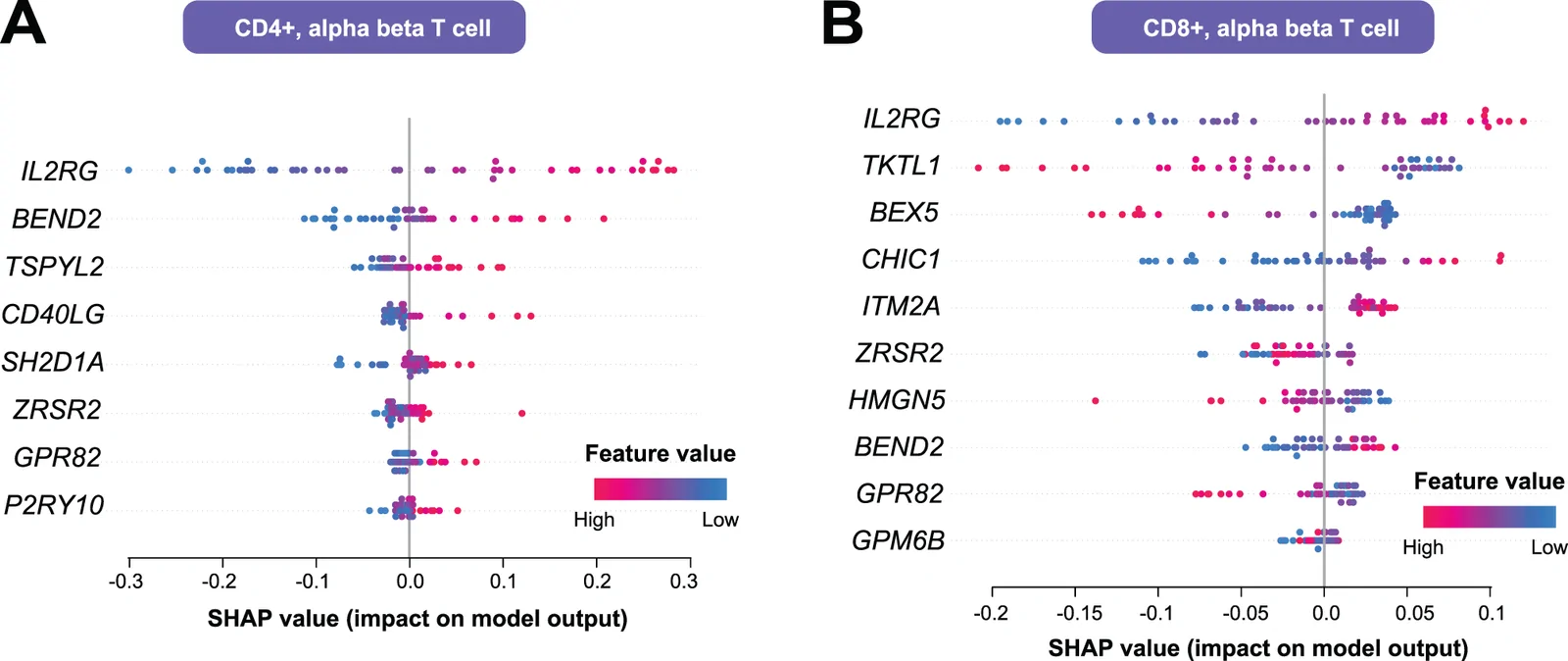
SHAP analysis of features in (**a**) CD4^+^ and (**b**) CD8^+^ T cells. *IL2RG* was the top feature in CD4^+^ T cells, while *IL2RG, TKTL1, BEX5,* and *ITM2A* contributed most in CD8^+^ T cells. Each point represents a sample; colour indicates feature value (blue = low, red = high), and position reflects SHAP value (feature impact on classification)

To interpret how individual genes influenced classifier decisions, we applied Shapley additive explanations (SHAP), a game theory-based approach that estimates the contribution of each feature to the final classification score [39]. SHAP values were calculated using a representative ensemble model retrained on the first data split, with hyperparameters averaged across partitions. In CD4^+^ T cells, *IL2RG* showed the strongest and most consistent positive contribution to SLE classification, with higher expression associated with higher classification probability of SLE. The remaining selected features showed the same direction of effect but with smaller magnitudes, consistent with the differential expression results (Fig. 3a). In CD8^+^ T cells, *IL2RG* again exhibited a strong positive influence, but the overall range of SHAP values was larger than in CD4^+^ T cells, indicating greater heterogeneity in how gene expression contributes to classification. *TKTL1* showed the opposite pattern, with lower expression driving the model outputs toward SLE, in line with its observed downregulation. Additional features, such as brain expressed X-linked 5 (*BEX5*) and integral membrane protein 2A (*ITM2A*), also displayed clear patterns with low *BEX5* expression and high *ITM2A* expression positively influencing SLE classification (Fig. 3b).

### CD8^+^ T cell model classifies SLE in an independent dataset

**Fig. 4:**
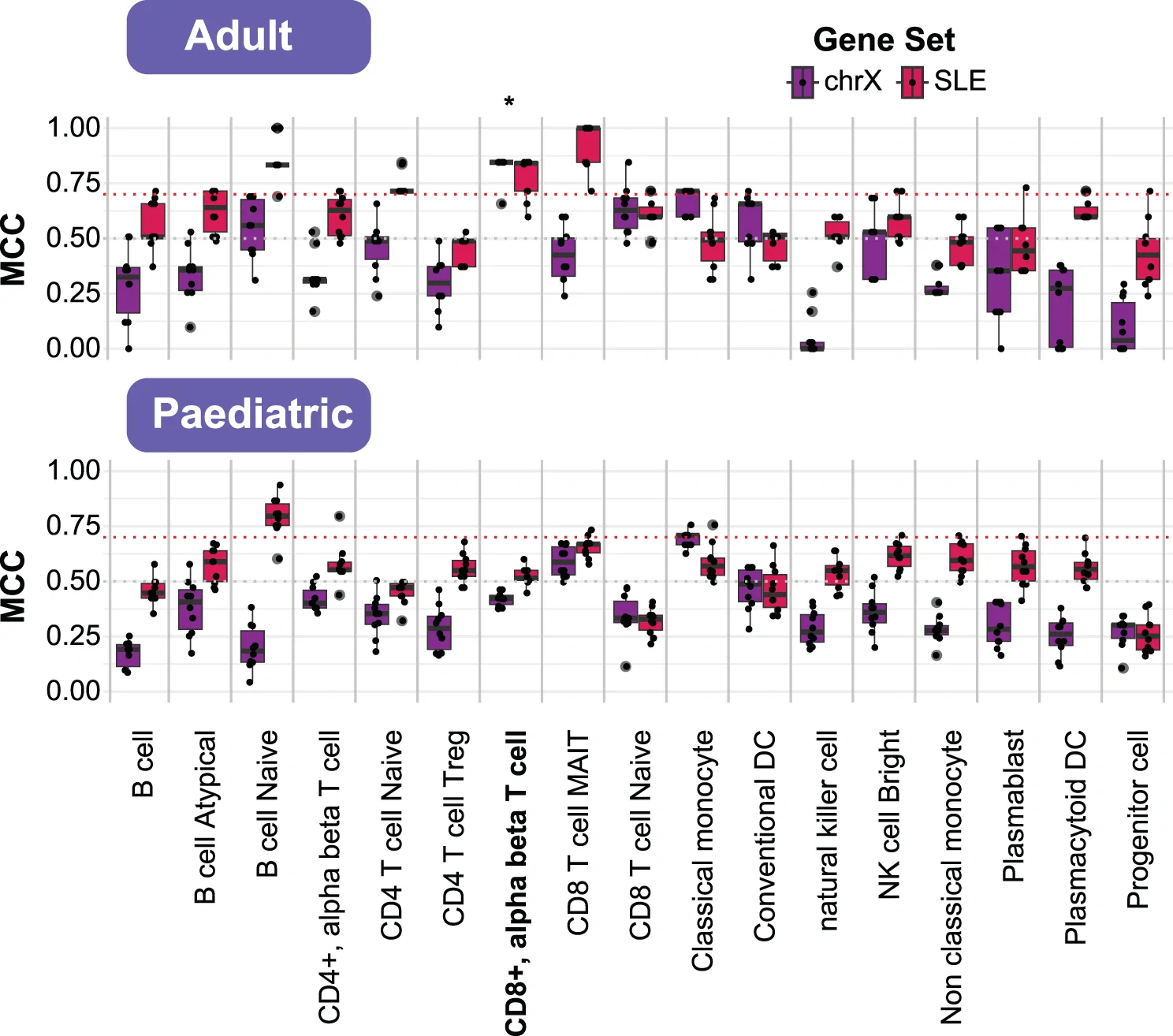
Generalisation of X chromosome and SLE gene models to an independent SLE dataset. Boxplots show the distribution of MCC scores for each immune cell type when ensemble models trained on X chromosome genes or SLE-associated genes classified an external scRNA-seq dataset of PBMCs from adult and paediatric SLE patients and healthy donors. MCC values were calculated separately for adult (top panel) and paediatric (bottom panel) cohorts. Boxplot centre lines indicate medians; box limits indicate interquartile ranges (IQR); whiskers extend to 1.5 × IQR. The red dashed line indicates MCCs above the 0.75 threshold. A result was considered replicable if both gene sets were above that line

A significant challenge in ML is overfitting to the training data, resulting in models that fail to generalise to unseen data. To assess generalisability, we applied our ensemble models to an independent scRNA-seq dataset of 310,310 PBMCs from 15 controls (10 paediatric and 5 adult) and 37 SLE cases (30 paediatric and 7 adult). Cell types were annotated using CellTypist [43] and harmonised with the labels used in the training dataset. Pseudobulked expression profiles were generated directly from raw counts without batch correction. As expected, both chrX and SLE models showed reduced performance when transferred to this independent cohort (Fig. 4). However, CD8^+^ T cells stood out in adults where chrX and SLE models performed similarly, with a 95% CI for MCC of 0.52–0.72 (Wilcoxon rank-sum test with FDR correction, *p* > 0.05). Notably, CD8^+^ T cells were the only population to retain a consistent and gene-set–independent classification signal across both datasets. This consistency is unlikely to arise from technical artefact alone, as the two datasets differ in donor age, sequencing chemistry, and batch correction. Instead, it suggests that CD8^+^ T cells carry a stable, reproducible X-linked transcriptional signature associated with SLE status that is preserved across adult cohorts. However, we do note that the sample size is small and further external validation would make this result more robust. Nevertheless, this finding points to the X chromosome as a source of generalizable, age-related, and cell-type–specific information in female SLE, particularly within CD8^+^ T cells.

### IL2RG expression is increased in T cells from patients with SLE

**Fig. 5:**
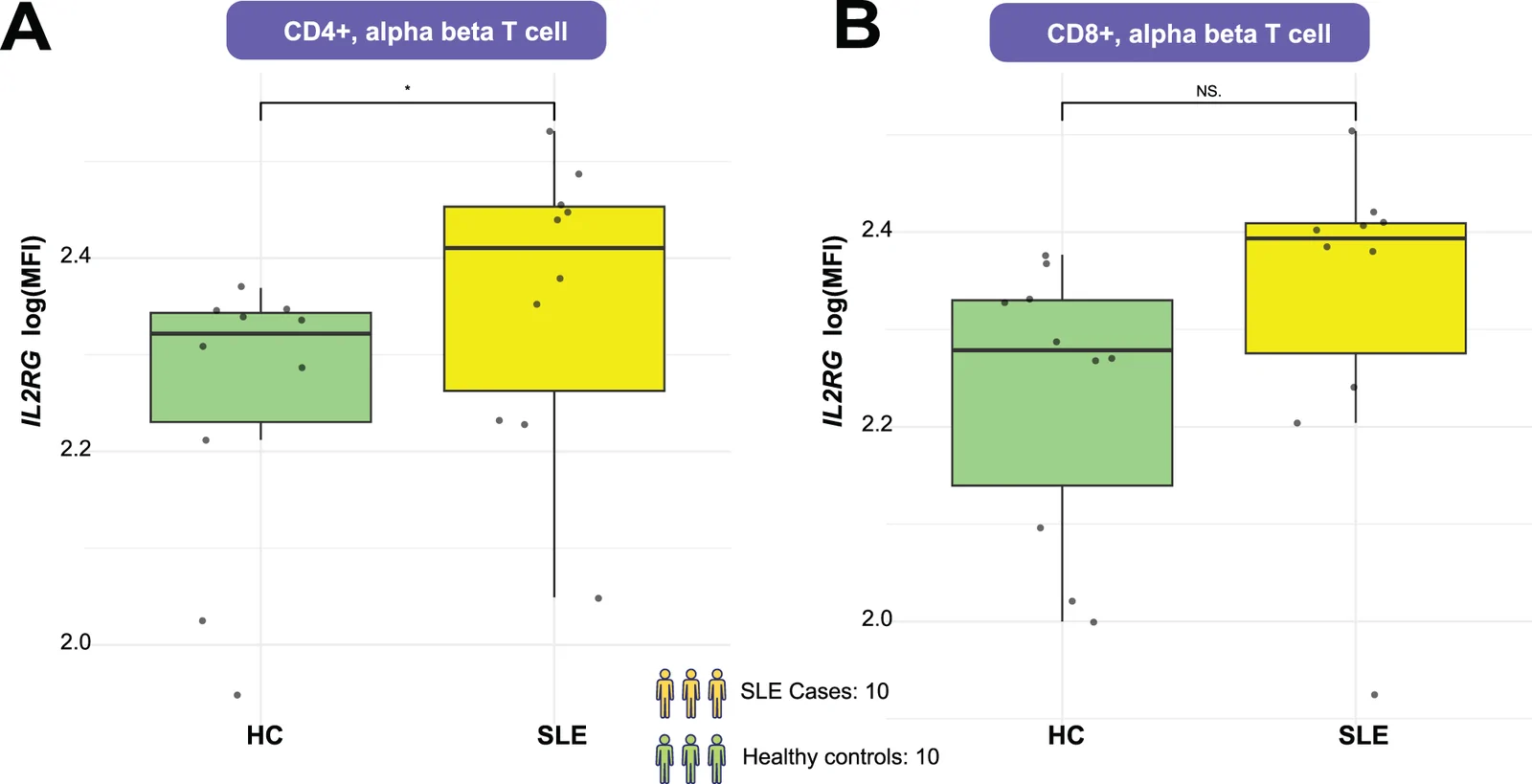
IL2RG expression is increased in T cells from patients with SLE. (**a**) IL2RG protein expression, measured as mean fluorescence intensity (MFI), in peripheral CD4^+^ and (**b**) CD8^+^ T cells from healthy controls (HC; *n* = 10) and patients with SLE (*n* = 10). IL2RG expression was significantly increased in CD4^+^ T cells from SLE patients (*p* = 0.043, Wilcoxon rank-sum test) and showed a trend toward elevation in CD8^+^ T cells (*p* = 0.052)

To validate our computational findings at the protein level, we quantified IL2RG protein expression in peripheral CD4^+^ and CD8^+^ T cells from an independent cohort of 10 female SLE patients, nine of whom were receiving immunosuppressive therapy at the time of sampling, and 10 healthy controls, using flow cytometry. IL2RG was selected for downstream validation based on its central biological role in cytokine signalling, its consistent importance across both CD4^+^ and CD8^+^ T cell models, and the feasibility of orthogonal validation due to its cell surface expression. IL2RG protein expression was significantly elevated in CD4^+^ T cells from SLE patients (*p* = 0.043, Wilcoxon rank-sum test) and showed a trend toward elevation in CD8^+^ T cells (*p* = 0.052) (Fig. 5a, b). However, given the low baseline expression of IL2RG and the predominance of immunosuppressed patients in the cohort, the observed differences likely reflect subtle shifts in gene dosage rather than large changes in protein abundance. Together, this finding supports the presence of subtle but detectable protein-level dosage effects consistent with an overall increase in expression, that could be linked to partial escape from XCI.

## Discussion

Using an ensemble of ML classifiers trained on X chromosome genes, we have identified features that accurately classified female SLE from controls, with XCI escape genes significantly overrepresented among features. These results are consistent with existing literature and reinforce the contribution of escape from XCI to the pathogenesis of SLE. We assessed the classification power of X chromosome models by comparing their performance to models trained on a set of literature-supported SLE genes. Within CD4^+^ and CD8^+^ T cells the comparable performance between X chromosome and SLE models suggests that the models learned an X chromosome transcriptomic signal for SLE classification. We then evaluated how overfit the models were to the training data by classifying an independent scRNA-seq dataset of paediatric and adult SLE. Here, CD8^+^ T cells indicate generalisability in the unseen adult cohort as the X chromosome model demonstrated consistent performance comparable to the SLE model. The reduced performance of the CD8^+^ T cell model in the paediatric cohort suggests that this X-linked signature may be an age-dependent phenomenon, rather than a congenital feature of SLE. Together with recent evidence that aging promotes reactivation of the inactive X chromosome, these data support a model whereby progressive erosion of XCI in lymphocytes contributes to female-biased autoimmune risk over time [50]. Recent work has shown extensive dysregulation of X-linked transcriptomes in SLE, including differential expression of *XIST* and genes of the *XIST*-interactome across adaptive and innate immune cells [51]. Whereas that study offered a comprehensive descriptive atlas of X-linked expression changes, our approach identifies a discriminatory subset of X-linked features that generalise across independent cohorts and highlight specific cell states, particularly within CD8^+^ T cells.

Moreover, this study offers a conceptual advance by indicating that changes in X chromosome expression are not simply secondary markers of inflammation but constitute a concentrated signal sufficient for accurate SLE classification. By focusing on the X chromosome, we move beyond characterising what is differentially expressed to determining the classification weight of these genes, establishing that X-linked transcriptional signatures are capable of classifying SLE.

Inspection of the features selected to train the CD4^+^ and CD8^+^ T cell models revealed X chromosome genes with known sex differences in their expression. For example, *IL2RG*, which emerged as a consistently informative feature in CD4^+^ and CD8^+^ T cells, encodes a cell surface receptor for the common gamma chain cytokines IL-2, IL-4, IL-7, IL-9, IL-15, and IL-21 [52]. Moreover, *IL2RG* expression was previously shown to be influenced by age and sex [53–55]. Recently, *Il2rg* was found to escape XCI in unstimulated and stimulated T cells from mice [19]. However, it is currently unclear whether *IL2RG* exhibits biallelic expression in human CD4^+^ and CD8^+^ T cells. In line with its selection in the classification models, IL2RG protein expression showed a subtle but significant increase in CD4^+^ T cells and a similar trend in CD8^+^ T cells from SLE patients. While limited by the detection sensitivity of flow cytometry for low-abundance surface proteins, this finding is consistent with the increased expression of *IL2RG* observed in the scRNA-seq data. Importantly, the inclusion of predominantly immunosuppressed SLE patients likely contributes to the modest effect sizes observed here. For example, a recent study reported that IL2RG (CD132) expression is significantly upregulated in lymphocytes from untreated SLE patients and positively correlates with disease activity, whereas this relationship is attenuated in treated cohorts [56]. The identification of *IL2RG* as a top feature across CD4^+^ and CD8^+^ T cells suggests a biological utility as a driver of the female-biased autoimmune response. This finding aligns with the recent development of a humanised monoclonal antibodies targeting CD132 (IL2RG) [56]. This highlights biological pathways that may be relevant for future therapeutic investigation. By ranking features by classification importance, our framework could be used to determine which X-linked changes represent the most promising candidates for future targeted interventions. Together, these results support the presence of subtle protein-level dosage effects consistent with increased gene expression. However, without further allelic expression information, we could not directly link it to partial escape of *IL2RG* from XCI, but it is a potential mechanism that needs to be further investigated.

Another gene CD40 ligand (*CD40LG*), selected in CD4^+^ T cells, is known to be hypomethylated on the inactive X chromosome in T cells from SLE patients [57]. Importantly, upregulated *CD40LG* in CD4^+^ T cells was demonstrated to stimulate the production of IgG autoantibodies by autologous B cells [58]. Transketolase like 1 (*TKTL1*) was the second highest ranked feature in CD8^+^ T cells and displayed a significant downregulation in SLE. *TKTL1* encodes an enzyme involved in glucose metabolism, with roles described in cancer biology. Although its functional role in SLE is unclear, its consistent selection by the models suggests it may contribute to the discriminative signal through subtle shifts in transcriptional programs associated with T cell activation states [59]. While *TKTL1* has not previously been associated with SLE, it was characterised as a variable escape gene [60]. The low SHAP scores for the remaining features suggest that classification performance may rely less on strong single-gene effects and more on the subtle, cumulative contribution of multiple features. This highlights a key advantage of our ML framework over conventional differential expression analysis. While differential expression identifies dysregulated genes in isolation, our model captures the synergistic interactions between features that individually may not reach significance thresholds. This incremental utility is particularly relevant for complex autoimmune diseases like SLE, where the cumulative effect of subtle gene dosage changes instead of single dominant drivers are likely to define the transcriptional state.

Furthermore, while we focus on CD4^+^ and CD8^+^ T cells, the strong classification performance observed across the Classical and Non-classical monocytes suggests that this X-linked transcriptional dysregulation may also be a feature of female SLE. The shared selection of XCI escape genes such as *CFP* and *TIMP1* within these innate subsets indicates that X-chromosome dosage defects may contribute to the broader inflammation driving SLE.

However, while the classification performance of the selected features supports the hypothesis of epigenetic dysregulation, we cannot definitively conclude that XCI escape or skewing is the sole driver of these transcriptional differences. Standard 3’ scRNA-seq lacks the allele-specific resolution required to confirm biallelic expression at the single-cell level. Therefore, alternative mechanisms must be considered to explain this X-linked signature, particularly within the highly inflammatory context of SLE.

For instance, the selection of *IL2RG* in our models may reflect altered IL-2 signalling pathways or broader shifts in T-cell activation states and subset composition, such as the expansion of activated CD8+ T cells. Furthermore, the chronic inflammatory environment in SLE is characterised by type-I interferon imprinting and the continuous, cytokine-driven activation of STAT signalling pathways [61, 62]. These systemic signals may upregulate the expression of specific X-linked immune genes independent of escape from XCI. Therefore, rather than resulting solely from dosage compensation failure, the predictive X-linked signature identified here likely represents a complex interplay between X-chromosome dysregulation and systemic inflammatory networks driving aberrant T-cell activation. Future allele-specific expression analyses are required to definitively untangle the contributions of physical XCI escape versus cytokine-induced transcriptional upregulation.

Our approach can be applied to any sufficiently large scRNA-seq dataset to identify biologically motivated gene sets that discriminate between conditions. Unlike traditional differential expression analysis, our approach can capture combined effects of multiple genes. However, interpretability remains a major challenge. While SHAP analysis highlighted the influence of individual features, the precise decision-making processes of ensemble ML models remain inaccessible. Diagnostically, the identification of a minimal but high-performing X-linked signature suggests that cost-effective assays might be explored to measure gene or protein expression in female patients. Our assessment of cell surface protein expression via flow cytometry supports this feasibility, as the technique is already a standard assay for clinical immunophenotyping. However, implementing these assays will require extensive validation in patient cohorts. Prognostically, the age-dependent decrease in classification performance observed in the paediatric cohort suggests that X-linked signatures may serve as biomarkers for disease progression or the transition from early-onset to adult-type SLE. Mechanistically, the selection of genes such as *IL2RG*, *CD40LG*, and *TKTL1* highlights cytokine signalling and immunometabolism pathways where X-chromosomal dosage effects may be most impactful, providing a focused roadmap for future functional validation.

To support the clinical utility of our X chromosome classification model, it is necessary to also consider established SLE biomarkers such as ANA and Type I IFN signatures. While ANA is highly sensitive for SLE screening, its low specificity remains a challenge in clinical practice. Conversely, while IFN signatures are hallmarks of SLE pathogenesis, they are often transient and fluctuate with disease activity. Our model provides a female-specific X chromosome signal that may provide complementary information to established biomarkers. We propose that an X-linked transcriptional signature could serve as a secondary stratification tool. In ANA-positive female patients, the presence of a high X-dysregulation score measured by upregulation of features like *IL2RG* and *CD40LG*, could distinguish SLE from other overlapping autoimmune or inflammatory conditions. Alternatively, this stratification may identify a subset of patients whose disease is specifically driven by X-linked dosage effects thereby warranting different therapeutic approaches. However, the potential to distinguish SLE from related conditions remains to be tested.

Several limitations should be acknowledged. First, XCI escape status could not be confirmed in the scRNA-seq data. Future studies should incorporate high-depth single-cell datasets enabling allele-specific expression analysis. This would allow for discrimination between true dosage compensation failure (i.e., XCI escape) and activation-induced upregulation. For example, a recent re-analysis of the Perez et al. (2022) dataset concluded that the power was insufficient to detect escape at single-cell resolution, although several candidates were suggested [20]. Second, the consequences of the identified expression differences for additional protein markers, including CD40LG and TKTL1, remain to be validated experimentally. Additional flow cytometry could assess their changes in expression. However, differences in protein-level within our validation cohort may be attenuated by patients undergoing immunosuppressive treatment. Therefore, we recommend future studies incorporate untreated cohorts. In addition, RNA fluorescence in situ hybridisation (RNA-FISH) targeting nascent transcripts should be explored as a validation strategy to directly visualise biallelic expression and confirm XCI escape at the single-cell level [63]. Third, differences between the training and validation cohorts, including sequencing chemistry, ancestry composition, and batch correction, likely contributed to reduced generalisation. Nevertheless, the reproducible performance of CD8^+^ T cells strengthen the confidence that the selected features capture a disease-relevant signal. Additionally, the absence of a well-known SLE X-linked gene *TLR7* in our results was due to the filtering criteria used in our analysis on gene expression levels. This is also consistent with the original Perez et al. work. Finally, due to low sample sizes the exclusion of patients experiencing active disease flares and those of Hispanic, Latin American, or African American ancestry, populations that often experience a higher disease burden and greater severity, represents a significant limitation that restricts the generalisability of these findings [64]. The features identified here may not fully capture the transcriptomic variation associated with flares or ancestry-specific risk factors. Therefore, our findings should be interpreted as a proof-of-concept for X-linked classification in managed female SLE rather than a model representative of the full, heterogenous real-world SLE population. This limitation highlights the urgent need for more diverse genomic cohorts to ensure that machine learning-derived diagnostics are equitable across the populations most in need. However, despite these limitations, we still observe strong signals of X-linked genes that could be used as female-specific proxies for XCI escape or dysregulation, providing a foundation for future work.

## Conclusions

In summary, this study shows that an ML-based approach identified X chromosome genes, many of which escape XCI, that classify female SLE with an accuracy comparable to established SLE-associated genes. These findings advance our understanding of the X chromosome’s contribution to the female bias in SLE pathogenesis and provide a framework for applying similar ML-based approaches to other autoimmune diseases. While our ensemble ML framework successfully identifies a robust X-linked signature in female SLE, we acknowledge that the exclusion of specific ancestries and active disease states limits the immediate clinical generalisability of these findings to broader, real-world populations. Future studies integrating allele-specific expression and protein-level validation will be essential for translating these classification signatures into mechanistic and therapeutic insights.

## Electronic supplementary material

Below is the link to the electronic supplementary material.

Supplementary Material 1

## Data Availability

The relevant code to reproduce the analyses and figures of this study is available via GitHub at https://github.com/ballouzlab/sle_ml_x/tree/main and 10.5281/zenodo.18853801.

## Abbreviations

ANA: Antinuclear antibodies
AUC: Area under the curve
AUPRC: Area under the precision recall curve
BEX5: Brain expressed X-linked 5
CD40LG: CD40 ligand
DEGs: Differentially expressed genes
FDR: false discovery rates
FN: false negatives
FP: false positives
FPR: false positive rate
GBM: gradient boosting machine
GEO: Gene Expression Omnibus
IFN: Interferons
IL2RG: interleukin 2 receptor gamma
IQR: interquartile range
ITM2A: integral membrane protein 2A
logit: logistic regression
MCC: Matthews Correlation Coefficient
MFI: mean fluorescence intensity
ML: machine learning
MLP: multilayer perceptron
NCBI: National Center for Biotechnology Information
PBMCs: peripheral blood mononuclear cells
pDCs: plasmacytoid dendritic cells
RF: random forest
ROC: receiver operating curve
scRNA-seq: single-cell RNA sequencing
SHAP: Shapley additive explanations
SLE: systemic lupus erythematosus
SVM: support vector machine
TKTL1: transketolase like 1
TLR7: toll-like receptor 7
TN: true negatives
TP: true positives
TPR: true positive rate
UMAP: Uniform Manifold Approximation and Projection
XCI: X chromosome inactivation
chrX: X chromosome

## Data availability

The datasets generated and/or analysed during the current study are available in the repository (10.5281/zenodo.18853801). All scRNA-seq data used in this study are available online from public databases. The raw and batch corrected counts from Perez et al. (2022) are available in the CellxGene database https://cellxgene.cziscience.com/collections/436154da-bcf1-4130-9c8b-120ff9a888f2 and GEO database under accession code GSE137029 (https://www.ncbi.nlm.nih.gov/geo/query/acc.cgi?acc=GSE137029). The raw counts from Nehar-Belaid et al. (2020) are available in the GEO database under accession code GSE135779 (https://www.ncbi.nlm.nih.gov/geo/query/acc.cgi?acc=GSE135779) [7].
